# Systems assessment of intercalated combination of chemotherapy and EGFR TKIs versus chemotherapy or EGFR TKIs alone in advanced NSCLC patients

**DOI:** 10.1038/srep15355

**Published:** 2015-10-20

**Authors:** Han Yan, Qin Li, Wei Wang, Hongchao Zhen, Bangwei Cao

**Affiliations:** 1 Department of Oncology, Beijing Friendship Hospital, Capital Medical University, Beijing, 100050, China; 2 Medical Healthcare Center, Beijing Friendship Hospital, Capital Medical University, Beijing, 100050, China

## Abstract

Both chemotherapy and epidermal growth factor receptor tyrosine kinase inhibitors (EGFR TKIs) are widely applied for the treatment of non-small cell lung cancer (NSCLC), but the efficacy of these two treatments in combination is not yet clear. Thus, we sought to evaluate the efficacy of the intercalated combination of these two treatments in NSCLC. The PubMed database, EMBASE, Cochrane Controlled Trials Register, and Chinese Biomedical Database were systematically searched by two researchers for randomized clinical trials (RCTs) that examined the intercalated combination of chemotherapy and EGFR TKIs in NSCLC. Ten studies involving 1,660 patients were included in this systematic review. The statistical results showed that the intercalated combination of chemotherapy and EGFR TKIs significantly improved overall survival (OS) (hazard ratio (HR) = 0.83, 95% confidence interval (CI): 0.70–0.98), progression-free survival (PFS) (HR = 0.65, 95% CI: 0.51–0.84), and the objective response rate (ORR) (risk ratio (RR) = 1.90, 95% CI: 1.22–2.98) compared to chemotherapy alone. Similarly, compared to EGFR TKIs monotherapy, the intercalated combination of chemotherapy and EGFR TKIs seemed superior to EGFR TKIs alone in terms of PFS, ORR and DCR (PFS: HR = 0.75, 95% CI: 0.62–0.91, ORR: RR = 1.49, 95% CI: 1.12–2.00 and DCR: RR = 1.33, 95% CI: 1.15–1.54) in advanced NSCLC therapy.

Non-small cell lung cancer (NSCLC) is the leading cause of cancer-related mortality worldwide. In the United States, approximately 224,210 new cases of lung or bronchial cancer were predicted to be diagnosed in 2015, and 156,260 deaths from lung cancer were predicted to occur in 2015^1^. Furthermore, the 5-year survival rate of lung cancer is only approximately 16.6%^2^. Unfortunately, in more than 50% of patients, this disease has already progressed to the advanced stage at the time of diagnosis, causing the opportunity for curative resection to be lost. Platinum-based cytotoxic doublet chemotherapy and molecular-targeted drugs are the main treatments for NSCLC, but the prognosis of advanced NSCLC remains poor. To achieve a better survival benefit for advanced NSCLC patients in clinical practice, platinum-based cytotoxic doublet chemotherapy combined with molecular-targeted agents has become the new focus of many investigations^3^,^4^.

Compared to conventional cytotoxic chemotherapy, epidermal growth factor receptor tyrosine kinase inhibitors (EGFR TKIs), such as gefitinib, erlotinib, and afatinib, have been confirmed to significantly prolong overall survival (OS) and progression-free survival (PFS) in advanced NSCLC patients who possess EGFR mutations^5^,^6^,^7^. Many clinical trials have demonstrated that EGFR TKIs have an excellent clinical survival benefit in advanced NSCLC patients, although traditional cytotoxic chemotherapy still plays an important role in the treatment of NSCLC^8^,^9^,^10^. To improve the survival of NSCLC patients, the combination of chemotherapy and EGFR TKIs was used in clinical treatment, but the results of many investigations have been controversial. As this is a novel treatment method, two patterns of treatment have been examined: chemotherapy and EGFR TKI administration synchronously (administration at the same time) or nonsynchronously (administration at alternating times). In the first method, four large-scale phase III randomized controlled trials (RCTs), including INTACT-1, INTACT-2, TALENT, and TRIBUTE, were performed in Europe and the United States since 2004 to evaluate if chemotherapy combined with either gefitinib or erlotinib synchronously as the first-line treatment for advanced NSCLC patients could improve survival^11^,^12^,^13^,^14^. Although these four RCTs included more than 4,000 participants, the results showed that EGFR TKIs combined with chemotherapy synchronously did not improve survival compared to placebo control. For the second method, which evaluated EGFR TKI oral administration between chemotherapy cycles, the results were inconsistent.

The FAST-ACT trial reported no significant differences in OS or tumor response rates between the chemotherapy-only group and the group of EGFR TKI administration between chemotherapy cycles^3^. However, the FASTACT-2 trial showed a prominent improvement in OS and PFS in the group that received chemotherapy and interval EGFR TKIs compared to the chemotherapy only group^15^. In addition, in a series of clinical trials, OS and PFS were diametrically opposed in the group of EGFR TKI administration between chemotherapy cycles compared to the EGFR TKIs alone group^16^,^17^. Based on the above clinical trials results, we sought to perform a systematic assessment to verify whether the intercalated combination of chemotherapy and EGFR TKIs is superior to chemotherapy alone or EGFR TKIs alone in the treatment of NSCLC.

## Results

### RCT identification and eligibility

**Figure 1:**
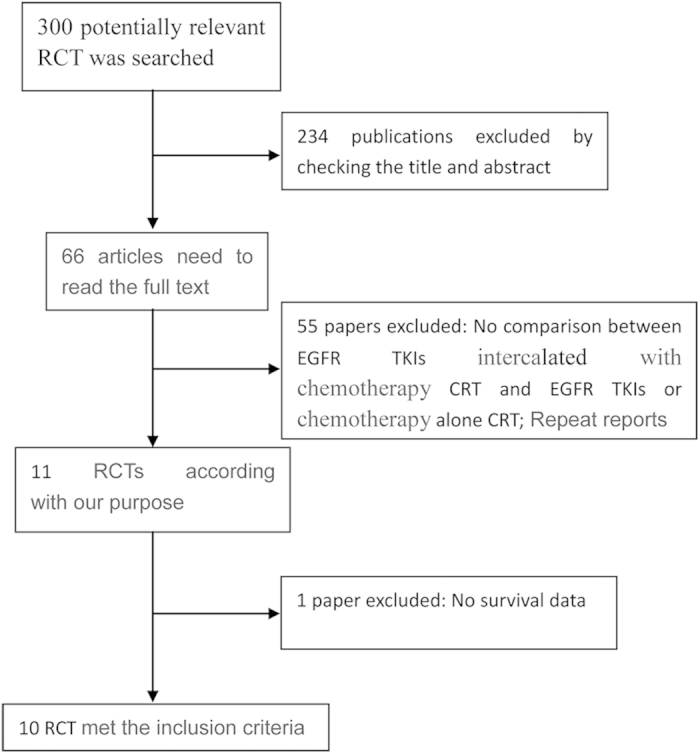
Flow chart of selection of RCTs for the Systems assessment.

Three hundred articles were obtained during the primary search (Fig. 1). By reading the title, abstract, and full text of each article, unrelated trials that did not meet the inclusion criteria were excluded. Ten RCTs^3^,^4^,^15^,^16^,^17^,^18^,^19^,^20^,^21^,^22^ with a total of 1,660 patients compared the intercalated combination of chemotherapy and EGFR TKIs to chemotherapy alone or EGFR TKIs alone and were found eligible for this systematic review (Table 1). In accordance with the Cochrane Handbook for Systematic Reviews of Interventions, the methodological qualities of each study were independently assessed by two authors and are presented in Fig. S1.

### The intercalated combination of chemotherapy and EGFR TKIs versus chemotherapy alone

**Figure 2:**
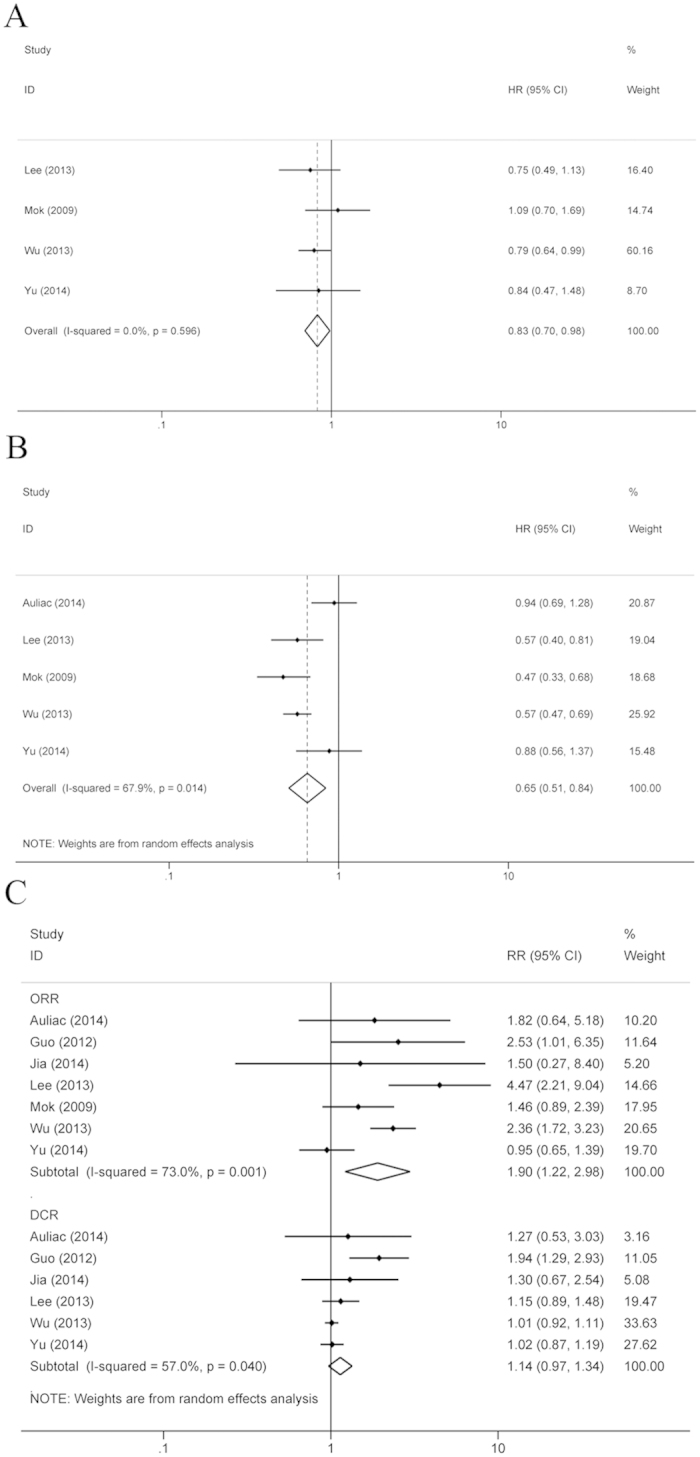
Compared the chemotherapy plus interval EGFR TKIs with chemotherapy alone. (**A**) OS (**B**) PFS; (**C**) ORR and DCR.

Seven RCTs with 1,166 patients performed a clinical benefit analysis in the group of EGFR TKI administration between chemotherapy cycles and the chemotherapy alone group. There was no heterogeneity between these studies (OS, I^2^ = 0%, P = 0.596; PFS, I2 = 0%, P = 0.638), so the fixed effect model (FEM) was used for data analysis. Compared to the chemotherapy alone group, the pooled hazard ratios (HRs) for OS and PFS in the group of EGFR TKI administration between chemotherapy cycles showed significantly reduced risks of death and disease progression (OS: HR = 0.83, 95% confidence interval (CI): 0.70–0.98, P = 0.027; PFS: HR = 0.65, 95% CI: 0.51–0.84, P = 0.001) (Fig. 2). The random effects model (REM) was used for the statistical analysis of objective response rates (ORRs) between the chemotherapy plus interval EGFR TKI group and the chemotherapy alone group, because the three RCTs showed heterogeneity (I2 = 73.0%, P = 0.001) (Fig. 2). Compared to chemotherapy alone, the statistical results showed that the ORR was significantly improved in the chemotherapy plus interval TKIs group (risk ratio (RR) = 1.90, 95% CI: 1.22–2.98, P = 0.005). The disease control rates (DCRs) of the two different treatment patterns were reported by six RCTs (Fig. 2), and there was heterogeneity between two studies (I2 = 57.0%, P = 0.040). The pooled RR for DCR showed that regardless of the treatment pattern used for NSCLC treatment, no significant difference existed between the two groups (RR = 1.14, 95% CI: 0.97–1.34, P = 0.116).

**Figure 3:**
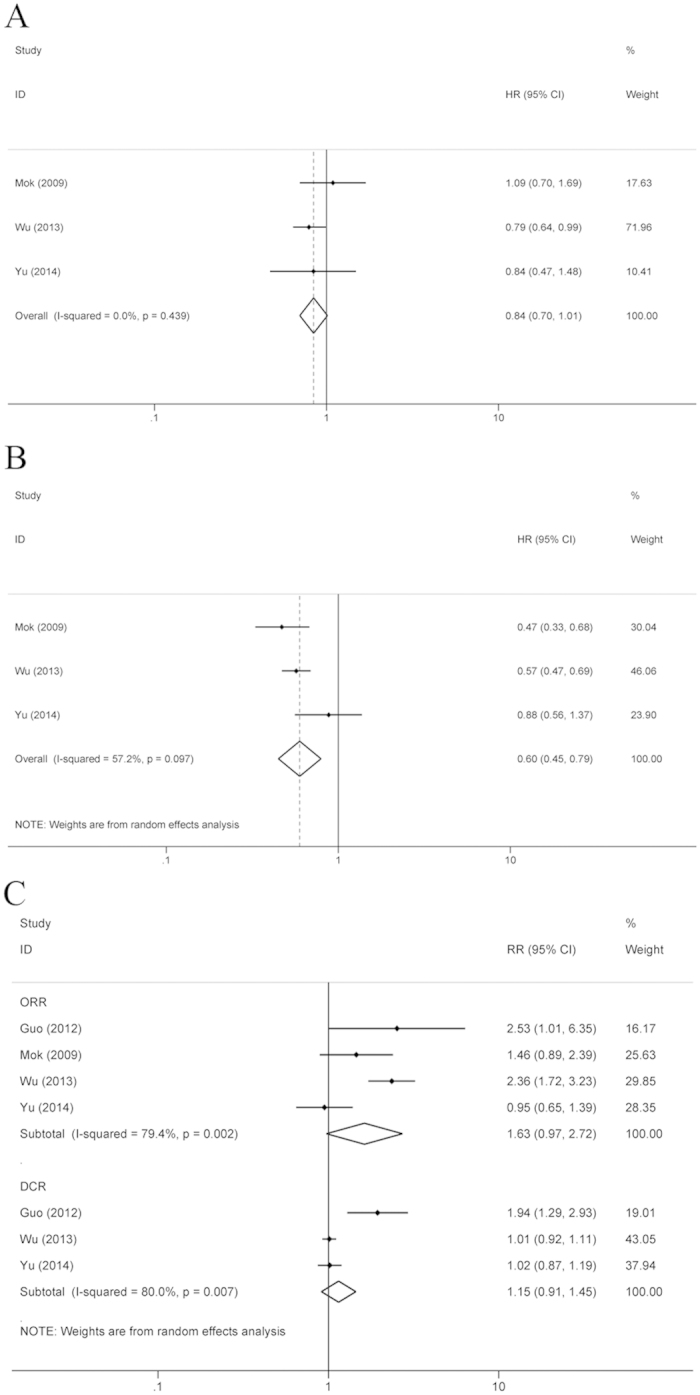
Compared the chemotherapy plus interval EGFR TKIs with chemotherapy alone as the first-line treatment. (**A**) OS; (**B)** PFS; (**C**) ORR and DCR.

For the first-line treatment of NSCLC, 3 RCTs reported the HRs of OS and PFS (Fig. 3). The risk of disease progression was significantly lower in the group of EGFR TKI administration between chemotherapy cycles compared to the chemotherapy alone group (HR = 0.60, 95% CI: 0.45–0.79, P < 0.001), but OS was not different between the two groups (HR = 0.84, 95% CI: 0.70–1.01, P = 0.068) (Fig. 3). Four RCTs presented data on ORR, which compared the intercalated combination of chemotherapy and EGFR TKIs to chemotherapy alone for the first-line treatment of NSCLC, and no difference in ORR was found (RR = 1.63, 95% CI: 0.97–2.72, P = 0.063) (Fig. 3). The data on DCR were available in three RCTs. The addition of EGFR TKIs to chemotherapy did not improve DCR for the first-line treatment of NSCLC (RR = 1.15, 95% CI: 0.91–1.45, P = 0.245) (Fig. 3).

### The intercalated combination of chemotherapy and EGFR TKIs versus EGFR TKIs monotherapy

**Figure 4:**
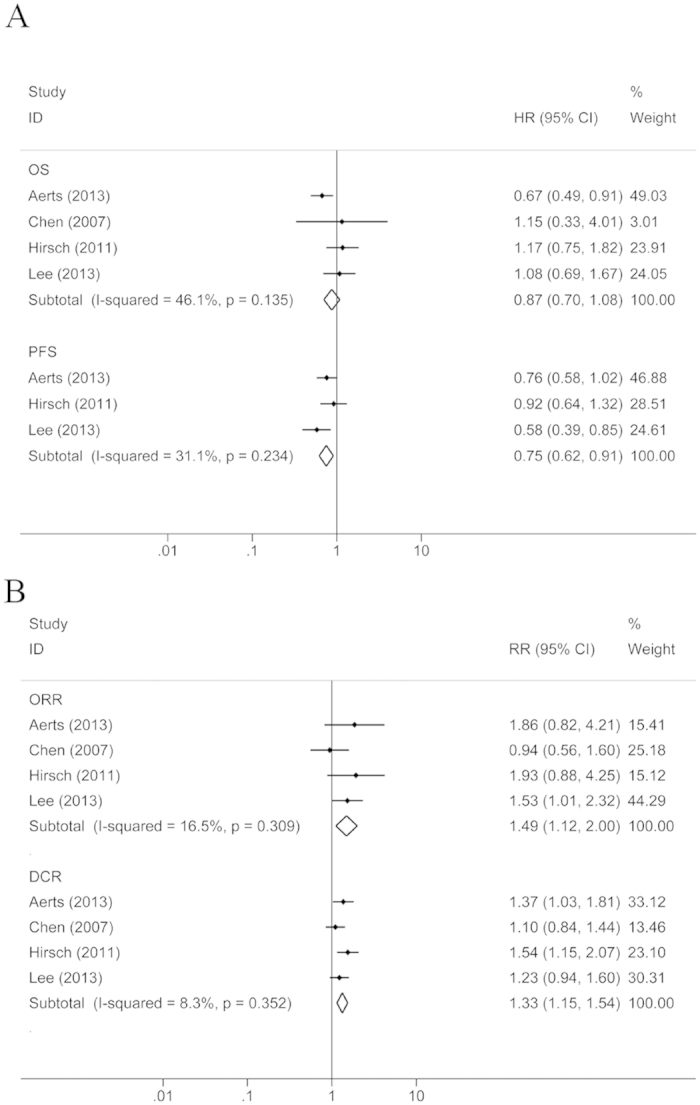
Compared the chemotherapy plus interval EGFR TKIs with TKIs monotherapy. (**A**) OS and PFS; (**B**) ORR and DCR.

Four RCTs with 575 patients were included in the clinical benefit analysis, which compared the group of EGFR TKI administration between chemotherapy cycles to the EGFR TKIs monotherapy group. There was no heterogeneity in OS or PFS among the four studies, so FEM was applied. Compared to the EGFR TKIs monotherapy group, there was no significant improvement in OS in the group of EGFR TKI administration between chemotherapy cycles (HR = 0.87, 95% CI: 0.70–1.08, P = 0.218), but PFS was significantly prolonged (HR = 0.75, 95% CI: 0.62–0.91, P = 0.004) (Fig. 4). Because there was no heterogeneity among the four RCTs, the FEM was applied in the analysis of ORR and DCR (Fig. 4). In the group of EGFR TKI administration between chemotherapy cycles, the ORR (RR = 1.49, 95% CI: 1.12–2.00, P = 0.007) and DCR (RR = 1.33, 95% CI: 1.15–1.54, P < 0.001) were significantly higher than in the EGFR TKIs alone group.

**Figure 5:**
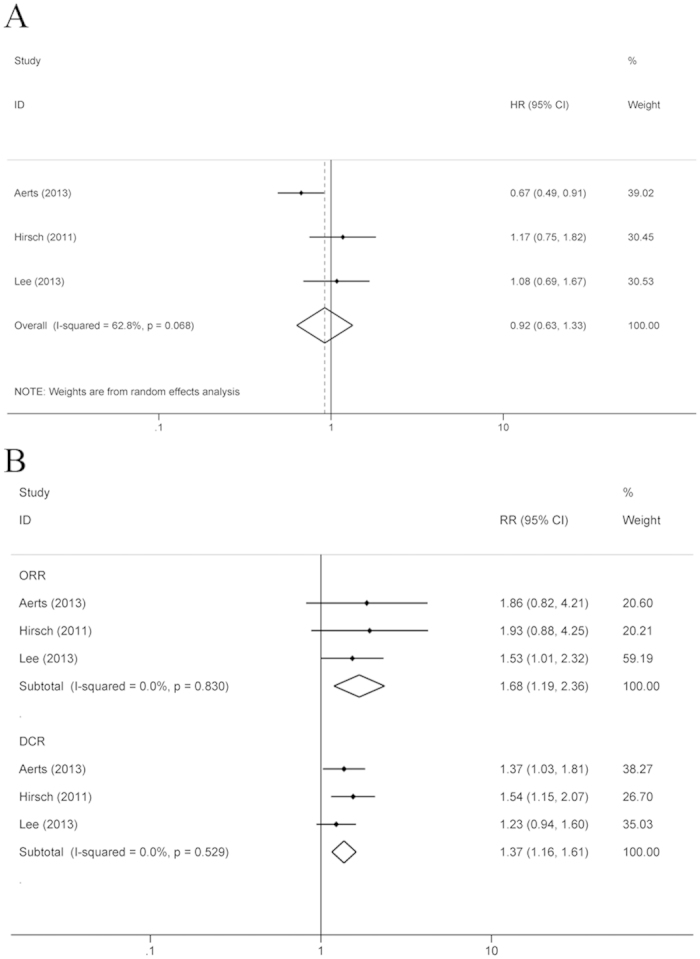
Compared the chemotherapy plus interval EGFR TKIs with TKIs monotherapy as the first-line treatment. (**A**) OS; (**B**) ORR and DCR.

For the first-line treatment of NSCLC, 3 RCTs reported the HRs of OS, ORR and DCR. ORR and DCR were higher in patients who received the intercalated combination of chemotherapy and EGFR TKIs than in patients who received EGFR TKI monotherapy (ORR: RR = 1.68, 95% CI: 1.19–2.36, P = 0.003; DCR: RR = 1.37, 95% CI: 1.16–1.61, P < 0.001), but no survival benefit of chemotherapy plus interval EGFR TKIs was found (HR = 0.92, 95% CI: 0.63–1.33, P = 0.656) (Fig. 5).

### Subgroup analysis and publication bias

**Figure 6:**
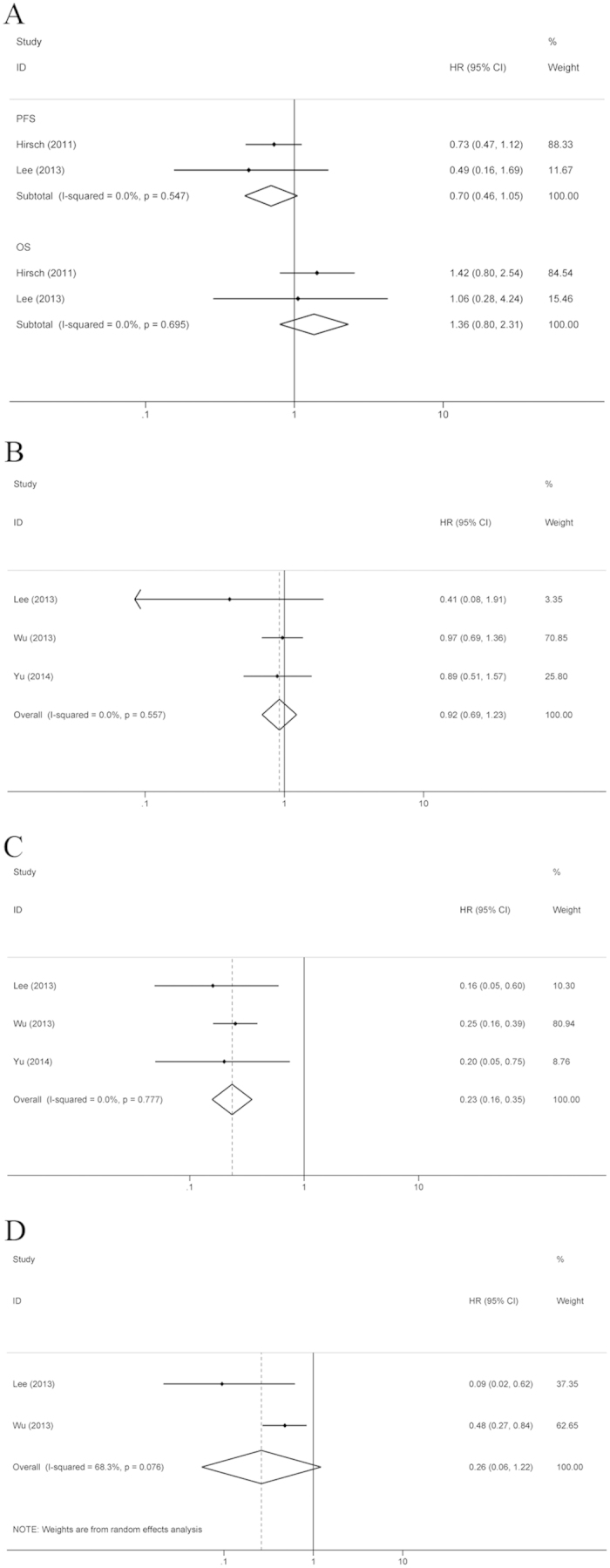
Subgroup analysis of EGFR genotype. (**A**) PFS and OS of chemotherapy plus interval EGFR TKIs vs. EGFR TKI monotherapy for the NSCLC patients with EGFR gene wild type; (**B**) PFS of chemotherapy plus interval EGFR TKIs vs. chemotherapy alone for the NSCLC patients with EGFR gene wild type; (**C**) PFS of chemotherapy plus interval EGFR TKIs vs. chemotherapy for the NSCLC patients with EGFR mutations; (**D**) OS of chemotherapy plus interval EGFR TKIs vs. chemotherapy for the NSCLC patients with EGFR mutations.

Of the ten RCTs, only three reported survival data under the two treatment patterns in NSCLC patients with wild-type EGFR. There was no heterogeneity in OS or PFS between the two studies which reported the data in that subgroup; therefore, FEM was applied for the comparison of the group of EGFR TKI administration between chemotherapy cycles and the EGFR TKI monotherapy group (OS, I^2^ = 0%, P = 0.695; PFS, I^2^ = 0%, P = 0.547); for the NSCLC patients with wild-type EGFR, OS and PFS were similar between these two treatment groups (OS, HR = 0.70, 95% CI: 0.46–1.05, P = 0.084; PFS, HR = 1.36, 95% CI: 0.80–2.31, P = 0.260) (Fig. 6A). For the comparison of the group of EGFR TKI administration between chemotherapy cycles to the chemotherapy alone group, because the data in the original literature were incomplete, only PFS was analyzed. There was no heterogeneity in PFS between the two studies, so FEM was applied (I^2^ = 0.0%, P = 0.557). The results revealed that regardless of the pattern of treatment for NSCLC patients with wild-type EGFR, no significant difference existed between the two groups (HR = 0.92, 95% CI: 0.69–1.23, P = 0.574) (Fig. 6B).

For the patients with EGFR mutations, only three trials reported the survival data of the group of EGFR TKI administration between chemotherapy cycles and the chemotherapy alone group, and data for the comparison between the combination therapy group and EGFR TKIs monotherapy group could not be analyzed. There was no heterogeneity in PFS between the three studies; therefore, FEM was applied (I^2^ = 0%, P = 0.777). The results showed that PFS was significantly prolonged in the chemotherapy plus interval TKIs group (HR = 0.23, 95% CI: 0.16–0.35, P < 0.001) (Fig. 6). For the statistical analysis of OS between the group of EGFR TKI administration between chemotherapy cycles and the chemotherapy alone group, because the two RCTs were found to possess heterogeneity (I^2^ = 68.3%, P = 0.076), the REM was used. For patients with EGFR mutations, OS was similar between the two groups (HR = 0.26, 95% CI: 0.06–1.22, P = 0.087) (Fig. 6). The potential presence of publication bias was evaluated by both the Begg’s test and Egger’s test; the results showed that no significant publication bias existed (Table 2).

## Discussion

This systematic review was conducted to compare the clinical efficacy of chemotherapy plus interval EGFR TKIs and chemotherapy or EGFR TKIs alone for the treatment of NSCLC. Up to Feb. 2015, the PubMed database, EMBASE, Cochrane Controlled Trials Register, and Chinese Biomedical Database were searched, and ten relevant RCTs with 1660 NSCLC patients were included in this systematic review. The results of this review showed that the intercalated combination therapy pattern, which involves the administration of EGFR TKIs between chemotherapy cycles, prominently increased the survival benefit of the treatment of advanced NSCLC. Compared to chemotherapy alone, the intercalated combination therapy significantly improved OS, PFS, and ORR in advanced NSCLC patients (P < 0.05). Similarly, the intercalated combination of chemotherapy and EGFR TKIs significantly prolonged PFS (HR = 0.74, P = 0.004) and ameliorated DCR (RR = 1.33, P < 0.001) relative to EGFR TKI monotherapy. For the first-line treatment of NSCLC, the intercalated combination of chemotherapy and EGFR TKIs showed improved PFS compared to chemotherapy alone and improved ORR and DCR compared to EGFR TKI monotherapy. To clarify whether EGFR gene mutation status affects clinical prognosis with these two treatment patterns, subgroup analyses were performed, and the results indicated that only the NSCLC patients who possessed an EGFR gene mutation acquired a survival benefit in terms of PFS (P < 0.05) with the method of EGFR TKI administration between chemotherapy cycles compared to chemotherapy alone.

Previous relevant studies showed that the combination of chemotherapy and EGFR TKIs was superior to chemotherapy alone^23^,^24^, but we believe that this benefit is mainly achieved with the intercalated combination treatment pattern compared with the synchronous combination pattern. In this study, the chemotherapy drugs that were used in combination with EGFR TKIs included pemetrexed, docetaxel, gemcitabine, vinorelbine, and paclitaxel, with or without platinum. The similar results of this study to those of previous studies supports the hypothesis that EGFR TKIs may act synergistically with chemotherapy agents. In cell line experiments, pemetrexed increased EGFR phosphorylation and reduced Akt phosphorylation, which ultimately enhanced the sensitivity of the tumor to EGFR TKIs. Furthermore, erlotinib was found to increase the expression of thymidylate synthase and, subsequently, enhance the sensitivity of tumor cells to pemetrexed. Paclitaxel and gefitinib showed similar molecular mechanisms^25^; however, other mechanisms that account for the synergy between cytotoxic drugs and EGFR TKIs are not yet clear, and further research is needed.

In conclusion, we found that the intercalated combination of chemotherapy and EGFR TKIs significantly improved OS, PFS, and ORR compared to chemotherapy alone for the treatment of advanced NSCLC and significantly improved PFS and ORR compared to EGFR TKI monotherapy. However, there are some limitations to this systematic review. In regards to patient selection, this study was not based on individual cases but, rather, was a pooled analysis of previously published data. Moreover, not all of the included studies provided EGFR mutation status and histological type. To obtain more convincing data, rigorous phase III clinical trials should be performed to further explore the potential benefits of chemotherapy combined with EGFR TKIs in advanced NSCLC patients.

## Methods

### Search method

This systematic review was performed in accordance with the guidelines of the Preferred Reporting Items for Systematic Review and Meta-analyses (PRISMA) statement^26^. The PubMed database (1966 to Feb. 2015), EMBASE (1974 to Feb. 2015), Central Registry of Controlled Trials (CENTRAL) and the Cochrane Library and the Chinese Biomedical Database (1978 to Feb. 2015) were searched (up to Feb. 2015). The initial search used the following MeSH terms: (lung neoplasms OR pulmonary neoplasm OR lung neoplasm OR pulmonary neoplasms OR lung cancers OR lung cancer OR pulmonary cancer OR pulmonary cancers) AND (gefitinib OR Iressa ORZd1839 OR erlotinib OR Tarceva ORCp-358774 OROSI-774 OR afatinib). To avoid the risk of selection or information bias, only RCTs were included in the analysis. Abstracts of the World Congress of Lung Cancer (2007–2014) and American Society of Clinical Oncology (2007—2014) were also searched.

### Inclusion criteria

The following five selection criteria were applied: 1) all studies were RCTs; 2) all patients had advanced NSCLC (III/IV) confirmed by histology or cytology; 3) when overlapping cohort studies were encountered, only the trial with the longest follow-up was included; 4) the enrolled trials at least provided data of OS, PFS, and time to disease progression (TTP); and 5) the experimental arm only received EGFR TKIs orally between cycles of chemotherapy, and the control arm received EGFR TKIs or chemotherapy alone. The screening of relevant articles was independently conducted by two researchers (Dr. H.Y. and Q.L.), and the qualities of the enrolled RCTs were assessed by Dr. HY according to the Cochrane Handbook 4.2.6 for Systematic Reviews of Interventions^27^.

### Data extraction

All identified abstracts were assessed independently by two investigators (H.Y. and Q.L.) in accordance with the pre-defined inclusion criteria. If only one investigator considered an abstract to be eligible, the full text of the article was retrieved and reviewed in detail by both investigators. Any discrepancy was resolved by an arbiter (B.W.C) or by contacting the authors of the original study. The following data were extracted from each article: first author, year of publication, clinical stage, number of cases, chemotherapy regimens, primary endpoint, OS, PFS, TTP, and ORR.

### Statistical analyses

This systematic review was performed using Stata software (Stata version 12.0, College Station, Texas, USA). Using Stata12.0 software, HRs were calculated to assess the overall effect of the treatments on PFS and OS. If the HR was <1.0, the death or disease progression of the combined therapy group was considered to exceed that of the monotherapy group. The Mantel-Haenszel procedure was used to estimate the RR of ORRs or adverse effects (AEs). If the RR was <1.0, the combined therapy group was considered to have a less effective ORR or less AEs than the EGFR TKI monotherapy or chemotherapy alone group. When the P-value of heterogeneity was <0.05 or I^2^ was >50%, the REM was used; otherwise, the FEM was used. Begg’s and Egger’s tests were used to evaluate the publication bias of these RCTs^28^,^29^.

## Additional Information

**How to cite this article**: Yan, H. *et al.* Systems assessment of intercalated combination of chemotherapy and EGFR TKIs versus chemotherapy or EGFR TKIs alone in advanced NSCLC patients. *Sci. Rep.* **5**, 15355; doi: 10.1038/srep15355 (2015).

## Supplementary Material

Supplementary Information

Supplementary Information

## Figures and Tables

**Table 1:** Characteristics of the eligible trials included in the systems assessment.

Author	Year	Phase	Country	Treatments of experimental and control group	No. ofpatients	CR+PR(%)	OS(m)	PFS(m)	TTP(m)
Auliac *et al.*(GFPC 10.02)^19^	2014	II	Global	docetaxel 75 mg/m^2^ d1, erlotinib 150 mg d2-16	73	12.30	6.5	2.2	—
				docetaxel 75 mg/m^2^ d1	74	6.60	8.3	2.5	—
Chen *et al.*^4^	2007	II	China	Vinorelbine 15 mg/m^2^ D1; gefitinib 250 mg/d, D2–14	21	52.38	23.4	—	12.8
				Gefitinib 250 mg/d	27	55.56	13.3	—	7.1
Guo *et al.*^20^	2012	II	China	gemcitabine1250 mg/m^2^ on days 1 and 8, cisplatin 25 mg/m^2^, gefitinib 250 mg/d days 10–24	36	36.10	12.1	7.3	—
				gemcitabine1250 mg/m^2^ on days 1 and 8,cisplatin 25 mg/m^2^	35	14.30	10.8	5.8	—
Jia *et al.*^22^	2014	II	China	pemetrexed 500 mg/m^2^ day 1 or docetaxel 75 mg/m^2^ d1, gefitinib 250 mg/d days 2–20	33	9.10	10.4	4.2	—
				pemetrexed 500 mg/m^2^ day 1 or docetaxel 75 mg/m^2^ d1,	33	6.45	7.9	3.3	—
Lee *et al.*^16^	2013	II	Global	Pemetrexed 500 mg/m^2^ D1; erlotinib 150 mg/d D2–14	78	44.74	20.5	7.4	—
				Pemetrexed, 500 mg/m^2^ D1	80	10. 00	17.7	4.4	—
				erlotinib 150 mg daily	82	29.27	22.8	3.8	
Mok *et al.* (FAST-ACT)^3^	2009	II	Asian Pacific	Gemcitabine 1250 mg/m^2^ D1 & 8; cisplatin 75 mg/m^2^ D1 or carboplatin AUC 5 D1; erlotinib 150 mg/d, D15–28	76	35.55	17.29	6.86	—
				Gemcitabine 1250 mg/m^2^ D1 & 8; cisplatin 75 mg/m^2^ or carboplatin AUC 5 D1	78	24.36	17.66	5.46	—
Yu *et al.*^21^	2014	II	China	pemetrexed 500 mg/m^2^ day 1,ciplatin 75 mg/m^2^ or carboplatin AUC = 5, gefitinib 250 mg/d days 3–16	58	50.00	25.4	7.9	—
				pemetrexed 500 mg/m^2^ day 1,ciplatin 75 mg/m^2^ or carboplatin AUC = 5	59	47.40	20	7	—
Wu *et al.* (FASTACT-2)^15^	2013	III	Asia	Gemcitabine 1250 mg/m^2^ D1 & 8; carboplatin AUC 5 or cisplatin 75 mg/m^2^ D1; erlotinib 150 mg/d D15–28	226	42.92	18.3	7.6	—
				Gemcitabine 1250 mg/m^2^ D1 & 8; carboplatin AUC 5 or cisplatin 75 mg/m^2^ D1	225	18.22	15.2	6	—
Hirsch *et al.*^17^	2011	II	Global	Paclitaxel 200 mg/m^2^; carboplatin AUC 6; erlotinib 150 mg, D2–15	67	22.38	11.43	4.57	—
				Erlotinib 150 mg/d	69	11.59	16.7	2.69	—
Aerts *et al.* (NVALT-10)^18^	2013	II	Netherlands	Erlotinib 150 mg D2–16; docetaxel 75 mg/m^2^ D1 or pemetrexed 500 mg/m^2^ D1	116	12.93	7.8	6.1	—
				Erlotinib 150 mg/d	115	6.96	5.5	4.9	—

**Table 2:** The systems assessment of chemotherapy plus the interval EGFR TKIs versus control group.

control group		Heterogeneity		HR/RR (95% CI)	Begg’s test		Egger’s test	
		*P* value	I^2^		Z	*P*	t	*P*
Chemotherapy	OS	0.389	0.0	0.82 (0.69–0.98)	1.02	0.308	0.69	0.561
	PFS	0.638	0.0	0.55 (0.47–0.64)	−0.24	1.000	0.60	0.589
	ORR	0.033	70.6	2.36 (1.41–3.97)	0.00	1.000	0.38	0.717
	DCR	0.351	0.0	1.04 (0.95–1.14)	0.75	0.452	2.02	0.113
EGFR TKIs	OS	0.135	46.1	0.87 (0.70–1.08)	0.34	0.734	0.96	0.436
	PFS	0.234	31.1	0.75 (0.62–0.91)	0.00	1.000	−0.29	0.821
	ORR	0.309	16.5	1.49 (1.12–2.00)	0.34	0.734	0.60	0.609
	DCR	0.352	8.3	1.33 (1.15–1.54)	1.02	0.308	3.52	0.072
