# Supplementary material for: Systems assessment of intercalated combination of chemotherapy and EGFR TKIs versus chemotherapy or EGFR TKIs alone in advanced NSCLC patients
Source: Sci Rep. 2015 Oct 20;5:15355. doi: 10.1038/srep15355 (PMC4611484; doi:10.1038/srep15355)

**Systems assessment** **of chemotherapy plus the interval EGFR TKIs versus chemotherapy or EGFR TKIs alone in advanced NSCLC patients**

Han Yan^1^; Qin Li^1^, Wei Wang^2^, Hongchao Zhen^1^, Bangwei Cao^1^

^1^Department of Oncology, Beijing Friendship Hospital, Capital Medical University, Beijing, China, 100050

^2^Medical Healthcare Center, Beijing Friendship Hospital, Capital Medical University, Beijing, China, 100050

Correspondence and requests for materials should be addressed to B.C. ( [oncology@ccmu.edu.cn](mailto:oncology@ccmu.edu.cn))


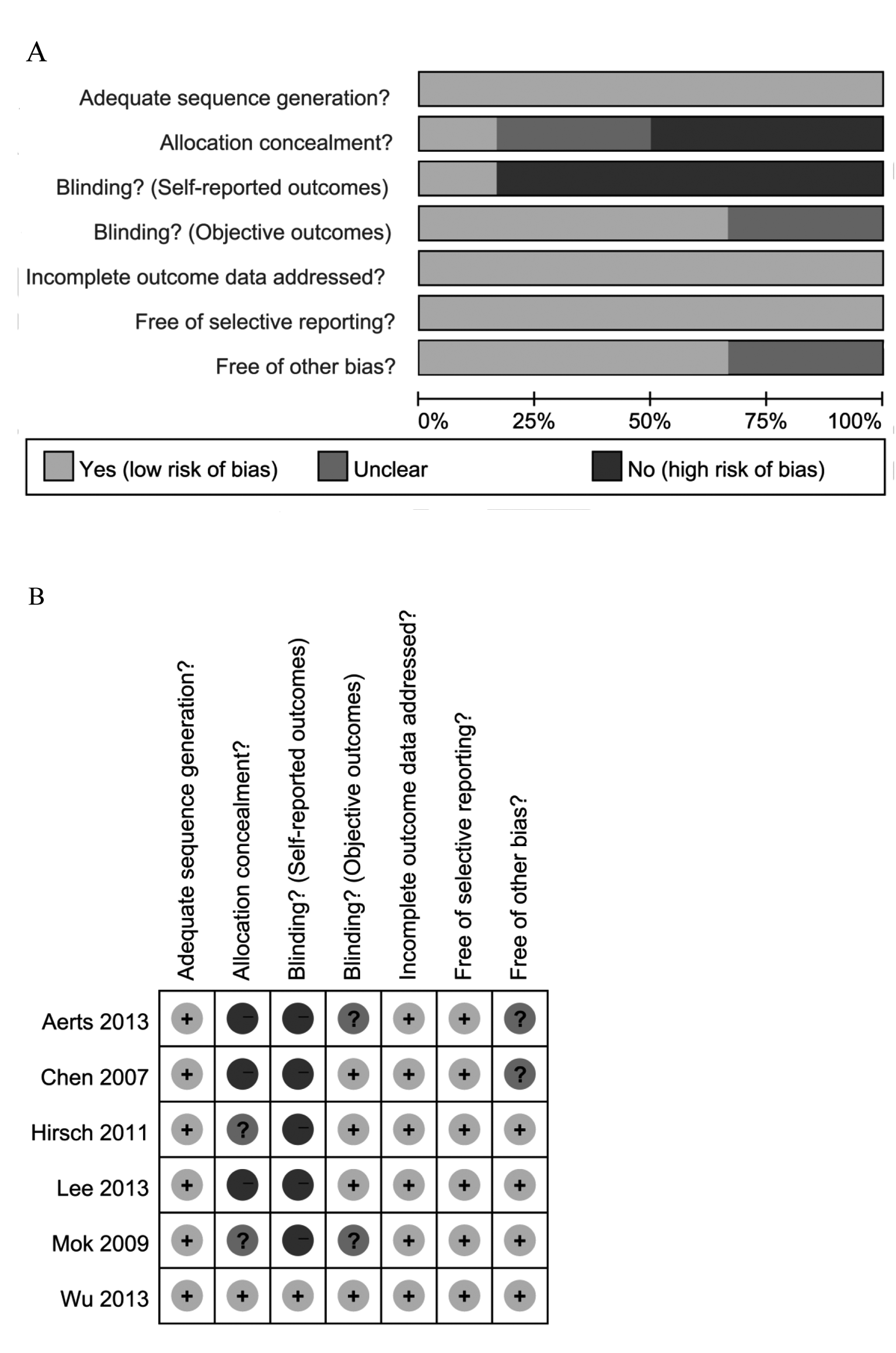

Supplement: Supplementary Information [file srep15355-s1.doc]
